# Live/Real Time Three-Dimensional Trans Esophageal Echocardiographic Findings in Amplatzer ASD Closure Devices in Adults

**Published:** 2012-09-15

**Authors:** Fatemeh Nabavizadeh, Navin C Nanda, Amitoj Singh, Carmen Mateescu

**Affiliations:** 1 Centre of Cardiology and Cardiovascular Sciences, Med Care Hospital, Dubai, UAE.; 2 Echocardiography labratories, University of Alabama at Birmingham, USA.; 3 Quality Coordinator, Canadian Specialist Hospital, Dubai, UAE.

**Keywords:** Atrial Septal Defect, Amplatzer Septal Occluder

## Abstract

Abstract

Six female patients aged from 19 to 73 years, with ostium secundum atrial septal defect underwent closure procedure with Amplatzer septal occluder device. Three-dimensional Echocardiography (3D-TEE) was done during the procedure or one day after the procedure. 3D-TEE provides incremental value over Two- dimensional trans-esophageal echocardiography in measuring Amplatzer septal occluder disc sizes and correlates well with manufacture device size. 3D-TEE will surely prove to increase the technical efficiency and it will become an important tool for the interventionists for periprocedural evaluation of device closures.

## 1. Introduction

Trans catheter closure has been accepted as an alternative to surgery in treatment of secundum type atrial septal defects (ASD) (1). Currently both ASD size and distance from important cardiac structures are considered in the selection of candidates for trans catheter closure (2). Amplatzer septal occluder is a self-expanding double disc nitinol mesh. It has two discs connected by a short waist .The waist size relates to the defect size (3, 4). Two types of ASD closure devices are available including Amplatzer septal occluder (ASO) which is indicated in Secundum ASD’s or patients who have undergone a fenestrated Fontan procedure and now require closure of fenestration. Patients indicated for ASD closure must have Echocardiographic evidence of ostium secundum ASD and clinical evidence of RV volume overload (i.e., 1.5:1 degree of left to right shunt or RV enlargement) (5). Another type is Amplatzer multifenestrated septal cribriform occluder indicated for closure of multifenestrated (cribriform) atrial septal defect (6, 7).

Device deployment position and success were evaluated by 2-D TEE or by periprocedural balloon stretch diameter described in other studies (3).

The aim of the present research was to perform pre and post procedural 3-D TEE as well to measure the device size and rims by 3D TEE, and compare the results obtained with the manufacturer’s device size, a study not reported previously.

## 2. Materials and Methods

Six female patients aged from 19 to 73 years, with ostium secundum ASD underwent Amplatzer septal occluder (ASO) device closure procedure. One patient, for which cribriform ASO was deployed, had aneurysmal fenestrated secundum ASD and the other five had secundum ASD.

3-D TEE was done during the procedure in five patients and one day after the procedure in one patient by Philips IE 33 Echocardiography machine, Data were recorded and then cropped in the X, Y, Z orthogonal planes and various oblique planes to obtain the 3D perspective.

**Figure 1.**
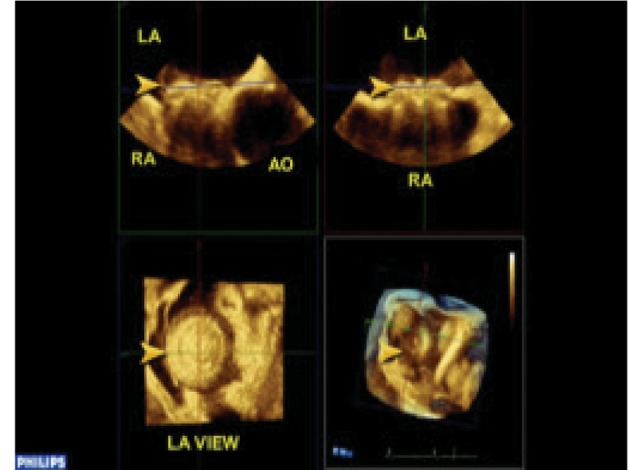
Size of LA Side of Device by QLAB.

Sizes of left atrial disc, right atrial disc and waist were measured by Q-LAB method (Figure 1). 

The results were compared with manufacturer’s left and right atrial discs, and waist size (Table 1). 

**Table 1:** Comparison between the Results Were and the Manufacturer’s Left and Right Atrial Discs, and Waist Size.

Age, Sex	Type Of ASD And Device	Observer [Table-fn fn5344]	ASD Measurements (Mm)	3 D Tee Measurements (Mm) From Device		ASD Device Size (By Manufacturer)	2D TEE Bubble Study	Residual Shunt On 3D TEE	Rim Size
			(BY 3D TEE)	LA[Table-fn fn5345]	RA	WAIST/LA/RA (mm)			
64,F*	Aneurysmal Fenestrated	A	NA	25.2	24.6				
	CribriformASO (25mm)	B		25.6	25.4	25 mm CribriformASO	POSITIVE	Positive One DayAfter Procedure	
						N/A/25/25			
53,F	Secundum ASD	A	17.4	34	30.2	20 mm ASO	Not Done	Negative	ASD-TV=13.4 mm
	ASO (20 mm)	B	19.9	34.8	30.8	4/34/30			
73,F	Secundum ASD	A	17.3	32.4	28.8	18 mm ASO	Not Done	Negative	ASD-AV=4.7 mm
	ASO (18 mm)	B	16.6	31.8	28.4	4/32/28			ASD-SVC=8.7 mm
									ASD-IVC=7.7mm
40,F	Secundum ASD	A	34.3	52.8	45.8	36 mm ASO	Not Done	Negative	ASD-AV=4.1 mm
	ASO(36 mm)	B	31.2	52.6	46.8	4/52/46			ASD-SVC=7.8 mm
	30.08 mm-BSD								ASD-IVC=8.6 mm
42,F	Secundum ASD	A	23.5	42.8	39.2	28mm ASO	Not Done	Negative	ASD-AV=4 mm
	ASO(28 mm)	B	28.2	42.6	38.6	4/42/38			
19,F#	Secundum ASD	A	34	54.6	47	38 mm ASO	Not Done	Negative	ASD-AV=3.2 mm
	ASO(38 mm)	B	32.6	54.4	48.4	4/54/48			ASD-SVC=8.5 mm

^a^A and B are two different readers to minimize inter observer error measurements.

^b^LA;Left Atrium; RA:Right Atrium

^*^In this patient due to fenestrated septum we did not measure ASD size before the procedure (Cribriform ASO device does not have a waist and has two equal discs). with cribriform ASO device both left and right atrial sides of the disc were equal and disc size was according to LA or RA side, whereas with other ASO devices LA side was larger than RA side and disc size was proportionate to the waist. In this patient the residual shunt disappeared on follow up.

^#^Malposition of ASD occluder was detected(over the tricuspid valve).Emergency removal of ASD occluder and patch repair of ASD with Bovine pericardium was done.

ASD distance from important rims including Aorta, Tricuspid valve, Superior vena cava, Inferior vena cava was measured. In one patient Bubble study was done by 2D TEE one day after the procedure, which showed leakage through the device, in the same patient 3-D TEE showed residual shunt and vena contracta of shunt(0.39 cm2) was measured by Q-LAB method 

**Figure 2.**
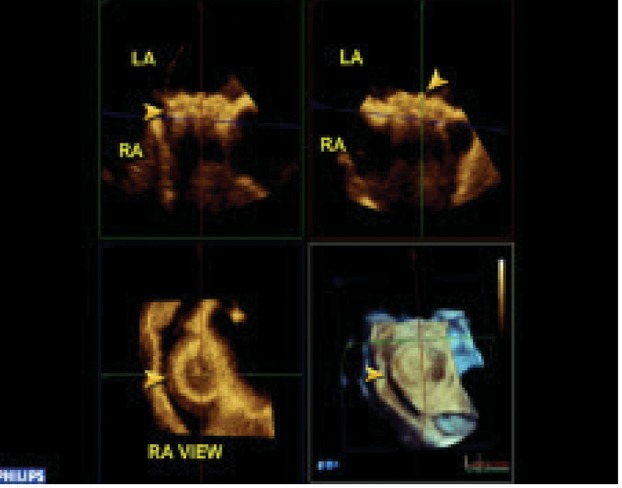
Size of Ra Side of Device by QLAB.

In one patient malposition of ASD occluder was detected over the Tricuspid valve for which ASD occluder was withdrawn immediately followed by patch repair of ASD with bovine pericardium. We could visualize enface view of both RA and LA discs. (Figure 1,2). 

## 3. Results

In all six patients we found very good correlation between manufacturer device size and device size measured by 3-D TEE.

In one patient with aneurysmal interatrial septum and cribriform ASO device, bubble study correlated with 3-D TEE result in showing residual shunt.( In this context, with cribriform ASO device both left and right atrial sides of the disc were equal and disc size was according to LA or RA side, but in regard to other ASO devices LA side was larger than RA side and disc size was proportionate to the waist). In this patient residual shunt disappeared on follow up echocardiography, as it was very tiny. Vena contrata of shunt through device was measured by Q-LAB method.

Enface view of both sides of discs, (not visible by 2-TEE), were easily seen by 3-D TEE.

The aspects related to the measurements from important rims including Aorta, IVC, TV, and SVC were as follows:

In one patient with aneurysmal ASD, no measurement could be obtained. However, ASD distance from important rims such as TV, AV, SVC, and IVC were determined in another patient with secundum ASD. In another four patients we could measure ASD distance from some of the important rims, and no comparison was made between the distance from rims and any other imaging modalities.

## 4. Discussion

As of September, 2007, a new three dimensional (3D)trans esophageal echocardiography (TEE) probe capable of live/real time 3-D imaging became commercially available for clinical practice in the United States (8).

This electronically steered transducer permits conventional multiplane two-dimensional (2D) TEE image acquisition, but also offers live 3D, 3D zoom, full volume 3D, and 3D color Doppler imaging, utilizing the matrix array technology employed in its 3D transthoracic echocardiography (TTE) predecessor (9).

Three –dimensional echocardiography has a long history. 3D TTE by the rotational method was reported in 1982 and 3D TEE in 1992, which largely remained a research tool due to the cumbersome reconstructive techniques required previously (9).

Since its initial description in 1976, Trans catheter closure of atrial septal defects (ASDs) has been performed employing varying closure techniques (5).

Currently, both ASD size and distance from important cardiac structures are considered in selecting candidates for attempted trans catheter closure (2). The defects with deficient IVC rim continue to be challenging. In addition, it is generally believed that the IVC rim cannot be consistently imaged through Trans esophageal echocardiography (2-D TEE) and Intra-cardiac echocardiography (ICE) is recommended to guide device deployment (10). Two–dimensional Transthoracic and Trans esophageal echocardiography have been used to assess these devices after placement, but they are of limited value because of their inability to visualize these devices in three dimensions (11).

Three-dimensional visualization of these devices may be potentially superior to two-dimensional echocardiography in the assessment of post-procedure complications such as presence and magnitude of residual shunt and device malposition, embolization, and fracture (12).

Color Doppler 3D TEE can show the residual shunt. This is a relatively common finding seen early after device implantation and tends to disappear with time (12).

The vena contracta could be cropped and measured, potentially providing more accurate assessment of the residual shunt (12).

Distance from important rims including IVC can be measured with the help of QLAB.

As further experience with 3D TEE will surely prove to increase the technical efficiency, we believe this new imaging modality will become an important tool for the interventionists for periprocedural evaluation of device closures.

As a complement to 2D examination, 3D TEE offers new views of adult congenital heart disease and has moved from a research tool into its rightful status.
